# Metabolomics of plant root exudates: From sample preparation to data analysis

**DOI:** 10.3389/fpls.2022.1062982

**Published:** 2022-12-06

**Authors:** Mohamed A. Salem, Jian You Wang, Salim Al-Babili

**Affiliations:** ^1^ Department of Pharmacognosy and Natural Products, Faculty of Pharmacy, Menoufia University, Menoufia, Egypt; ^2^ The BioActives Lab, Center for Desert Agriculture, Biological and Environment Science and Engineering (BESE), King Abdullah University of Science and Technology, Thuwal, Saudi Arabia; ^3^ Plant Science Program, Biological and Environmental Science and Engineering Division, King Abdullah University of Science and Technology (KAUST), Thuwal, Saudi Arabia

**Keywords:** root exudates, rhizosphere, metabolomics, sampling, multivariate analysis

## Abstract

Plants release a set of chemical compounds, called exudates, into the rhizosphere, under normal conditions and in response to environmental stimuli and surrounding soil organisms. Plant root exudates play indispensable roles in inhibiting the growth of harmful microorganisms, while also promoting the growth of beneficial microbes and attracting symbiotic partners. Root exudates contain a complex array of primary and specialized metabolites. Some of these chemicals are only found in certain plant species for shaping the microbial community in the rhizosphere. Comprehensive understanding of plant root exudates has numerous applications from basic sciences to enhancing crop yield, production of stress-tolerant crops, and phytoremediation. This review summarizes the metabolomics workflow for determining the composition of root exudates, from sample preparation to data acquisition and analysis. We also discuss recent advances in the existing analytical methods and future perspectives of metabolite analysis.

## Introduction

The term “rhizosphere” is commonly used to describe the soils modified by plant roots and the area around a plant root (plant-root interface) that is inhabited by a population of several organisms (Hartmann et al., 2008). The rhizosphere is one of the most significant hotspots of the plant ecosystem, determining nutrient acquisitions and pathogens control (Kuzyakov and Razavi, 2019). The chemicals released by plant roots in the exudates, include high and low molecular weight compounds from diverse chemical classes, such as amino acids, organic acids, alcohols, polypeptides, sugars, phenolics, enzymes, proteins, and hormones (Baetz and Martinoia, 2014). The amount and nature of the released exudates are influenced by many factors, such as plant taxa, age, root morphology, climatic conditions, nutrient availability, and biotic factors, e.g. soil microorganisms, herbivorous attacks or other neighboring plants (Sasse et al., 2018).

Since chemical compounds released in the root exudates shape the interaction between the plant and the surrounding environment, comprehensive metabolomics studies of plant root exudates are of great importance for basic science as well as for applications in enhancing crop resilience and improving stress tolerance (Escolà Casas and Matamoros, 2021). Albeit the progress that has been recently made in plant metabolomics, comprehensive analysis of a metabolome in the rhizosphere is still challenging. Despite the growing number of reviews demonstrating potential plant-microbiome interaction (Jacoby and Kopriva, 2019; Trivedi et al., 2020; Pang et al., 2021; Gupta et al., 2022; Trivedi et al., 2022), sampling root exudates (Oburger and Jones, 2018; Pantigoso et al., 2021), and analysis of exudates (Van Dam and Bouwmeester, 2016; Canarini et al., 2019; Escolà Casas and Matamoros, 2021), relatively few reviews have attempted to integrate the strategies for metabolomics analysis of plant root exudates from sample preparation to data analysis.

**Figure 1:**
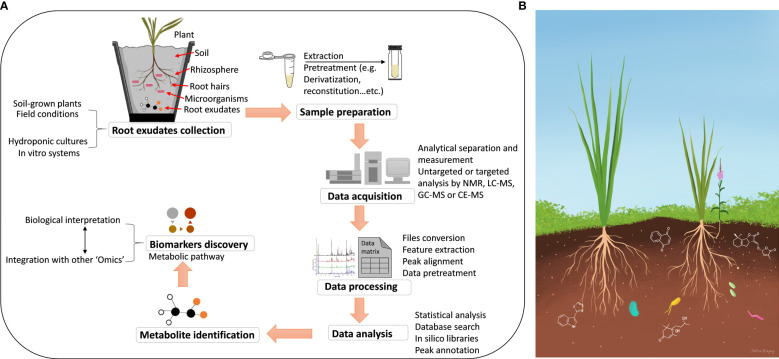
**(A)** Schematic representation of collection and high throughput analysis of metabolites secreted by plant roots. **(B)** Illustration of specialized compounds in the root exudates for organismal communications. The flowering plant represents the root parasitic weed *Striga hermonthica* growing on cereal roots.

Thus, this review presents a concise overview for metabolomics methods used in studying the chemistry of root exudates (**Figure 1A**). We discuss the existing sampling methods and extraction and describe how extracted samples are subjected to analysis using different analytical techniques. Moreover, we highlight the most commonly used methods that are based on hyphenated analytical methods, such as chromatography, either liquid chromatography (LC) or gas chromatography (GC), coupled to mass spectrometry (MS), as well as nuclear magnetic resonance (NMR). We also cover data mining strategies that are required for processing and evaluation. Finally, we discuss data analysis by multivariate statistical approaches for biological interpretation.

## Overview of root exudates in organismal communications

Living organisms could generally shape their local biotic environment. Indeed, plants utilize their root exudate to modulate nearby growth conditions, change the soil environments, recruit beneficial microbes for their survival, and communicate with microorganisms (**Figure 1B**) (Lugtenberg and Kamilova, 2009). Although many aspects of this process are still arguable (Reinhold-Hurek et al., 2015), numerous lines of evidence have reported the ability of the plant microbiome to regulate plant growth and development in response to different stresses (Lugtenberg and Kamilova, 2009). Moreover, plant root exudates also positively impact plant development and enhance fitness in response to plant pathogens (Yuan et al., 2018; Mavrodi et al., 2021). On the other hand, more than 40% of the carbon fixed during photosynthesis is released by plant roots in the form of exudates, secretions, and lysates (Badri and Vivanco, 2009), with highly variable compositions depending on plant species and environmental conditions (Phillips et al., 2004; De-La-Peña et al., 2008). Primary metabolites include sugars, amino acids, polypeptides, proteins, carboxylic acids, and fatty acids, have been well-characterized in the exudates of plant roots, which essentially serve as vital carbon, nitrogen, and energy sources for competitive root colonization and suppression of pathogenic soil microorganisms (Kamilova et al., 2005; Lugtenberg and Kamilova, 2009). Apart from supporting microbial proliferation, these exudate metabolites are also responsible the formation of distinct microbial assemblages in the rhizosphere (Berendsen et al., 2012).

Besides primary metabolites, countless secondary/specialized metabolites, generally overlooked compounds with significant bioactivities, are present in the root exudates (Jacoby and Kopriva, 2019; Pang et al., 2021), functioning as stimulants, inhibitors, or signaling molecules (Baetz and Martinoia, 2014; Fiorilli et al., 2019). For example, benzoxazinoids released by maize roots act as important herbivore and pathogen resistance factors and trigger rhizosphere colonization by the bacterium *Pseudomonas putida* (Neal et al., 2012; Pétriacq et al., 2017). In addition, root released camalexin and coumarins can increase plant growth and positively affect root microbiota under nutrient-limited conditions (Koprivova et al., 2019; Harbort et al., 2020). In addition, the carotenoid-derived plant hormones strigolactones (SLs) are important rhizosphere signals in the communication with arbuscular mycorrhizal (AM) fungi (Fiorilli et al., 2019; Ito et al., 2022), while the AM symbiosis is also regulated by several carotenoid-derived signaling molecules, such as zaxinone and blumenols (Wang et al., 2018; Wang et al., 2020; Wang et al., 2021). Notably, the biosynthesis and/or exudation of these specialized metabolites are highly correlated to nutrition deficiency, especially phosphate and nitrogen, suggesting the beneficial functions of plant-microbe interactions (Fiorilli et al., 2019; Wang et al., 2020; Ablazov et al., 2022; Votta et al., 2022). However, SLs are also used by root parasitic plants, such as *Striga* that causes up-to 10 billion yield losses in Africa (Jamil et al., 2022), to localize a suitable host and coordinate their germination with its presence (Wang et al., 2022). The dependency of obligate root parasitic plants on host released SLs has been recently used to control these weeds by inducing their seeds germination in the absence of the host by applying SL analogs, a promising strategy referred to suicidal germination (Jamil et al., 2020; Jamil et al., 2022). These examples highlight the importance of secondary metabolites in rhizosphere organismal communications and the need for more research to uncover and understand the ecological function of released specialized metabolites. This knowledge could very useful for engineering root exudate compositions to attract beneficial microbiome, improve pathogen defense and enhance tolerance to abiotic stress (Li et al., 2021; Zhou et al., 2022).

## Sample preparation

Plant metabolomics methods have been used for identifying primary and specialized metabolites in root exudates. Several existing methods for collection of root exudates have been described and reviewed (Oburger and Jones, 2018; Vives-Peris et al., 2020; Pantigoso et al., 2021; Wang et al., 2022). However, the collection approaches vary substantially among studies due to limitations in the accessibility of roots or experimental scales. The method used for root exudates collection depends on the objective of the study, such as SL research (Wang et al., 2022). Sampling can be done either from soil-grown plants or hydroponic culture systems. In soil, the chemical composition of root exudates usually varies due to adsorption on soil particles. Further, it can be altered when subjected to microbial degradation (Vives-Peris et al., 2020). In addition, the plant exudation profile is also dynamic and influenced by the composition of the surrounding microbiome (Oburger and Jones, 2018).

One of the main advantages of *in vitro* cultures over field conditions is the higher reproducibility, which is required for crucial studies on the effects of abiotic stress factors, including nutrient availability, temperature, moisture content, and osmotic status (Vranova et al., 2013). While the samples are obtained, in case of *in vitro* cultures, from a hydroponic system or liquid medium, collection of samples from plant grown in soil requires the use of sorption filters buried in the ground close to roots (Haase et al., 2007). Methods to collect leachate samples from greenhouse pots have also been established (Zhu et al., 2016; Pantigoso et al., 2021). Collection of root exudates from *in situ* in natural growing environment can lead to partial disruption of roots during collection, inducing stresses and causing sample contamination from the rhizosphere and the collection of metabolites that are not specific to the target exudation profile (e.g., microbial metabolites) (Vranova et al., 2013; Pantigoso et al., 2021). Additionally, changes in the physical characteristics of the soil can lead to severe changes in the root exudation profile (Sasse et al., 2018; Miller et al., 2019).

One of the major disadvantages of the *in vitro* cultures is the interference from the exogenously supplemented nutrients and ions in analysis. This has been avoided in some studies through collection of exudates from plants immersed in distilled water (Badri et al., 2013b). Further, one of the major limitations of *in vitro* grown plants is that the morphology and physiology of the roots is different from soil-grown plants, due to the lack of natural conditions such as soil clods and microorganisms (Vranova et al., 2013; Miller et al., 2019; Wang et al., 2019). To mimic plant’s natural environment in soil, sterile artificial soil mixtures (e.g. silica sands) suspended in liquid medium have been introduced more than 50 years ago (Harmsen and Jager, 1962; Curl and Truelove, 1986). The introduction of synthetic soil particles provides mechanical forces simulating natural settings (Vranova et al., 2013). To better simulate soil environment, microorganisms have been also introduced to the *in vitro* growing systems. Novel methods for non-destructive *in situ* and *in vitro* collection of root exudates have also been introduced and improved (Phillips et al., 2008; Gao et al., 2018). Spatial dynamics of the root exudates is quite challenging to analyze, however, soil-based sampling approaches of unaltered soluble exudates from the whole root system include using Soil-Hydroponic-Hybrid as well as rhizoboxes in combination with a root exudate collecting tool (SOIL-REC) (Oburger et al., 2014; Canarini et al., 2016; Oburger et al., 2022). To better discriminate target-specific metabolites from the root exudation profile, the utilization of isotope labelled substrate (e.g. ^13^CO_2_ incubation to quantitatively trace the photosynthesis-assimilated carbon) has been comprised to obtain labelled exuded metabolites (Seto et al., 2014; Simon and Haichar, 2019; Chen et al., 2022).

Since the exudation profiles differ depending on plant development, the targeted stage should be carefully defined prior to the collection of the root exudates (Chaparro et al., 2013). The plants should be removed from growth media, rinsed with water to get read of adhering media and transplanted in water for a pre-defined time (Badri et al., 2013a; Ray et al., 2018; Jin et al., 2019; Wang et al., 2019). The collected samples are pooled to have enough biological replicates, and the final solution is filtered and subjected to analysis. Sampling root exudates in salt solutions can result in high background matrix, particularly, when sample concentration is necessary prior to analysis (Oburger and Jones, 2018). Thus, sampling root exudates in water reduces the effect of salts. However, sampling in distilled water can damage the root cell membranes, altering the exudate concentrations. This can be reduced by submerging the roots in water solution for few minutes prior to final sampling (Oburger and Jones, 2018). In addition, by using reverse phase C_18_ silica columns for sample collection can also reduce the salt content (Wang et al., 2022). Further, filtration (through membrane filters, < 0.45 or 0.2 μm) or centrifugation is done to remove root debris. The samples can be concentrated to remove excess water through freeze-drying (lyophilization), while enough biological replicates should be considered (Salem et al., 2020). The inclusion of quality control (QC) samples, such as pooled samples allows for following metabolite recovery and technical errors during sample preparation and analysis (Broadhurst et al., 2018; Martins et al., 2018).

## Analytical methods for metabolite profiling of root exudates

Traditional photochemistry methods for extraction, fractionalization, purification and identification of pure chemical compounds require tens of kilograms of plant material in a laborious and time consuming process (Salem et al., 2020). Thus, these methods might not be suitable for labor sensitive and high throughput analysis of hundreds to thousands of metabolites in root exudates. Plant metabolomics represents large scale analysis of all metabolites within biological samples (Salem et al., 2020). Plant metabolomics methods have been used for identifying diverse metabolites for basic and applied research.

The most widely used methods in plant metabolomics include gas chromatography coupled to mass spectrometry (GC-MS), liquid chromatography-MS (LC-MS) and nuclear magnetic resonance spectroscopy (NMR). Other techniques such as capillary electrophoresis-MS (CE-MS), Fourier transform-near-infrared (FT-NIR) spectroscopy, MS imaging (MSI), and live single-cell mass spectrometry (LSC-MS) were also reported (Pang et al., 2021). Combination of the aforementioned methods may provide complementary information for in-depth analysis of metabolites. Since MS- and NMR-based detection methods are the most frequently used techniques in recent years, we summarize their applications, advantages and disadvantages in **Table 1**. Besides MS and NMR, non-destructive physical methods such as FT-NIR spectroscopy can also be quick, cheap, and easy to use method for the identification of functional groups in specific metabolites. However, further optimization in sample preparation is indispensable for better spectral quality (Dias et al., 2016). Further, MSI-based techniques were utilized to directly depict the spatial distribution of metabolites in the rhizosphere of *Zea mays* L. plants, *Brachypodium distachyon* roots, Arabidopsis seedlings, Asparagus roots, tomato roots and *Marchantia polymorpha* gemmalings (Sasse et al., 2020; Korenblum et al., 2020; Döll et al., 2021; Gomez-Zepeda et al., 2021; Lohse et al., 2021). Additionally, LSC-MS allows for the detection of hundreds of plant-specific metabolites acquired from a single plant cell, discriminating them from those produced by soil microbes (Masuda et al., 2018; Taylor et al., 2021).

**Table 1:** The key differences between the most frequently used techniques in plant metabolomics.

Techniques	Metabolites coverage	Advantages	Disadvantages
**GC-MS**	Thousands of metabolites, mainly, volatile organic compounds, fatty acids, sugars, nucleotides, organic acids and amino acids	▪ Sensitive for volatile metabolites ▪ Availability of standard libraries for identification ▪ Relatively inexpensive instrumentation ▪ High reproducibility	▪ Insensitive for thermo- labile metabolites ▪ Analysis is destructive in nature ▪ Derivatiztion is necessary for non-volatile metabolites ▪ Matrix and Ionization dependent response ▪ Novel compound identification is difficult
**LC-MS**	Thousands of most organic and some inorganic metabolites, particularly, secondary metabolites	▪ Sensitive for thermo-labile and polar metabolites ▪ Superior for targeted analysis ▪ High number of detected metabolites ▪ Direct injection without separation is possible	▪ Affected by high salt content ▪ Analysis is destructive in nature ▪ Limited structural information ▪ Many detected metabolites often remain unidentified ▪ Less reproducibility and robustness
**NMR**	Hundreds of metabolites, from most organic classes	▪ Minimal sample preparation ▪ Independent of matrix effects ▪ Powerful in structural elucidation ▪ Identification of novel compounds ▪ Non- destructive in nature ▪ Permits *in vitro* and *in vivo* flux analysis ▪ Precise quantification	▪ Less sensitivity than MS ▪ Peak overlap ▪ Expensive instrumentation ▪ Structural elucidation is very complex

## Data processing and analysis

Metabolomics experiments generate very complex data, the vast majority of which are derived from biological significance, while others from sample processing, background noise and contaminations also contribute to the obtained data (Duan and Qi, 2015). MS-based analysis is the most technical and conventional platform used for large scale analysis of metabolites, but it is not the most reliable for structural confirmation when compared to NMR (Salem et al., 2020). The raw data obtained from plant metabolomics studies are not suitable for direct analysis and need to go through processing strategies to obtain pure spectra including noise filtering, smoothing, deconvolution, peak alignment (Duan and Qi, 2015; Tahir et al., 2019). Processing of MS raw data for metabolomics analysis requires several steps, including file format conversion for the vendor-dependent binary format (e.g.wiff,.D,.RAW … etc.) into other common formats (e.g. NetCDF, mzML, mzXML … etc.) for further processing. Several open source and commercial software and web-tools are currently available for MS (e.g. XCMS, MZmine, MSDIAL, Open MS, Decon2LS, …etc.) data processing (Salem et al., 2020). Data pretreatment strategies such as scaling and normalization reduce the systematic bias, while maintaining the biological variation. Data scaling aims at minimizing the impact of dimension differences (e.g. different concentration of metabolites) giving all variables the same weighting, reducing the bias and improving the predictive ability of statistical models (Gromski et al., 2015).

Identifying marker metabolites and their biological significance is achieved through in-depth multivariate and univariate data analysis strategies (Gorrochategui et al., 2016; Rosato et al., 2018). To generate an overview of the relationship among the datasets, unsupervised analysis is a type of multivariate machine learning algorithm that attempts to analyze a dataset without prior knowledge of sample grouping. Principal component analysis (PCA) and hierarchical cluster analysis (HCA) are the most extensively used multivariate unsupervised statistical methods. Additionally, multiple machine learning algorithms have been developed for supervised analysis including partial least squares (PLS) regression, PLS-Discriminant Analysis (PLS-DA), linear discriminant analysis (LDA), K-nearest neighbor analysis (KNN), random forests (RF) and artificial neural networks (ANN) that contribute to the identification of potentially significant marker metabolites. Several open source as well as commercial web tools and softwares, such as MetaboAnalyst, Cytoscape, SIMCA (Umetrics) and SPSS (IBM), offer comprehensive metabolomics data analysis, visualization, and biological interpretation (Peters et al., 2018).

The complex and diverse chemistry of plant metabolites makes the identification process very challenging. Further, several plant specialized metabolites have no commercially-available standards, and also many have not been recorded by spectral databases. Most of the existing metabolomics databases do not contain a satisfying proportion of plant metabolites. The metabolite annotation, according to the Metabolomics Standards Initiative (MSI) (Sumner et al., 2007; Blaženović et al., 2018), is divided into four levels: 1) the first level: confidently identified compounds based on co-characterization with authentic samples; 2) the second level: putatively annotated compounds based on spectral similarity with spectral libraries; 3) the third level: putatively characterized compound classes, and 4) the fourth level: unidentified or unclassified unknown compounds. Although plant metabolites are diverse in their chemistry, they are composed of basic structural units with different substitutions e.g. flavonoids, fatty acids, alkaloids, terpenoids, and coumarins. Compounds that originate from the basic building unit will produce similar fragmentation pattern, allowing the determination of compound class and deduction of the substitution (Perez De Souza et al., 2020).

In GC/MS, several mass spectral libraries of standard compounds have been established. In addition, the chromatographic behavior and retention time can be converted to a more robust retention index giving an additional parameter for structural identification. Huge number of libraries for GC-MS analysis is available, including Golm Metabolom Database, Wiley, NIST, and Fiehn GC-MS library (Vinaixa et al., 2016). Standard mass spectra libraries for LC-MS are limited, and the metabolite identification is more dependent on the availability of authentic standards. Thus, metabolite identification is mainly based on chromatographic pattern of the target class, isotope pattern-assisted prediction of the molecular formula as well as MS^n^ fragmentation pattern. Searching using various databases such as PubChem, ChemSpider, MassBank, HMDB, KEGG, NIST, WILEY, METLIN, MoNa, mzCloud, GNPS and ReSpect, among others, provide information about the possible structures (Blaženović et al., 2018). For the annotation of unknown molecules, *in silico* fragmentation tools such as MS-FINDER, MetFrag, CFM-ID and CSI : FingerID are recommended (Blaženović et al., 2018). Further, bioinformatics tools based on molecular networking, such as GNPS, are powerful in structural elucidation of known and novel compounds of interest based on spectra similarity (Quinn et al., 2017; Perez De Souza et al., 2020).

Diverse databases provide detailed information about metabolomics data collected from multiple platforms along with searching, visualization, downloading tools and/or implementing biological relationships between metabolites through metabolic pathways. Examples are Plant Metabolome Database (PMDB), KNApSAcK, Metaboanlyst, PlantCyc database, Metabolomics.jp, KEGG, BioCyc, among others (Rosato et al., 2018). Recent advances in bioinformatics tools and other computer-aided approaches play a central part in systems biology approaches (Kumar et al., 2017). The concept of integrated omics is gaining much attention in the last decade as a System Biology tool for unraveling the holistic molecular perspectives of the complex biological processes (Pinu et al., 2019). Metabolites are directly closer to the phenotype than genes and proteins; thus they directly reflect the biochemical pathways. Different approaches for multi-omics data integration alongside their limitation have been extensively reviewed (Fondi and Liò, 2015; Misra et al., 2019; Pinu et al., 2019).

## Concluding remarks: Challenges and future perspectives

Metabolites from root exudates play important roles in mediating organismal interaction and response to environmental stresses. Studies have revealed that root-released metabolites can shape the root microbiome, and in turn, the microbiome has an impact on the host plant metabolome. However, little is known about the temporal and spatial dynamics of the root exudate profile. Root exudation is mostly analyzed from hydroponic cultures due to the chemical complexity of soil. Hence, analysis of metabolites from root exudates under normal physiological conditions with more natural settings is necessary. The choice of sampling strategy, sampling duration, time of collection, plant type as well as plant developmental stage will ultimately have a great impact on the obtained exudation profiles. The recent developments in the analytical approaches and methods in the metabolomics field have increased our understanding of the chemistry of root exudates. Several techniques such as GC-MS, LC-MS, CE-MS, FT-NIR, and NMR have been used for analysis of different classes of metabolites secreted by plant roots. Distinguishing plant-specific metabolites from those produced by associated microbes is a great challenge in analysis. However, the recent analytical developments such as mass spectrometry imaging (MSI), matrix-assisted laser desorption ionization (MALDI), laser ablation electrospray ionization (LAESI) and live single-cell mass spectrometry (LSC-MS) allowed metabolite analysis at a single-cell level, enabling the discrimination of plant-specific metabolites from those produced by associated microbiomes.

Aside from the recent advancements in metabolite data acquisition, several bioinformatics tools have been developed for peak annotation, statistical analysis, multi-omics data integration and potential molecular biomarker discovery. Though these improvements, the annotation of a whole metabolome is still challenging. A key bottleneck in metabolite characterization is the progress of metabolite annotation. Next, the strategies for integration of metabolomics with other omics approaches have not provided in-depth understanding of molecular interactions. Thus, the development of further machine learning computational approaches such as neural networks is stilled needed. Generating specialized databases for the chemistry of the root exudates is also important.

The current studies obtained so far will help us for better understanding of plant-microbiome interactions and should shape the future for crop breeding and sustainable crop production. However, many biological questions remain to be answered for deeper insights into the role of root exudates in shaping the organismal communications. For example, which species of the microbiome are attracted by a specific metabolite? How the combination of primary and specialized metabolites shape the plant microbiome? What are the dynamic changes of exudate metabolites at different developmental changes? Which classes of plant metabolites are secreted for attracting beneficial microbiome and which ones repel pathogenic microbiome? Why different plant species attract different microbiomes? Which classes of released metabolites are consumed by microbes? Answering these questions will significantly increase our knowledge about the ecological and physiological aspects of this largely overlooked aspect in plant’s life and may allow us to engineer the microbiome and increase plant’s pathogen defense, towards improving crops performance and decreasing the ecological and economic costs of agriculture.

## Author contributions

MAS, JYW and SA-B conceived, structured, and finalized the manuscript. All authors contributed to the article and approved the submitted version.
